# Supplementary material for: Accessing mental health services for a child living with anxiety: Parents’ lived experience and recommendations
Source: PLoS One. 2023 Apr 5;18(4):e0283518. doi: 10.1371/journal.pone.0283518 (PMC10075395; doi:10.1371/journal.pone.0283518)
Supplement: S1 File — (DOCX) [file pone.0283518.s001.docx]

**S1 File. Consolidated criteria for reporting qualitative studies (COREQ) checklist.**

Developed from:

Tong A, Sainsbury P, Craig J. Consolidated criteria for reporting qualitative research (COREQ): a 32-item checklist for interviews and focus groups. IJQHC. 2007;19(6): 349 – 357.

| **Item** | **Guide Question/Description** | **Reported on page #** |
| --- | --- | --- |
| **Domain 1: Research team and reflexivity** |  |  |
| *Personal Characteristics* |  |  |
| 1. Interviewer/facilitator | Which author/s conducted the interview or focus group? | p.8, line 201 |
| 2. Credentials | What were the researcher’s credentials? E.g. PhD, MD | p.8, lines 201-203 |
| 3. Occupation | What was their occupation at the time of the study? | p.8, line 201 |
| 4. Gender | Was the researcher male or female? | p.8, line 201 |
| 5. Experience and training | What experience or training did the researcher have? | p.8, lines 201-203 |
| *Relationship with participants* |  |  |
| 6. Relationship established | Was a relationship established prior to study commencement? | p.8, lines 203-204 |
| 7. Participant knowledge of the interviewer | What did the participants know about the researcher? e.g. personal goals, reasons for doing the research | p.8, lines 203-204  lines 205-207 |
| 8. Interviewer characteristics | What characteristics were reported about the inter viewer/facilitator? e.g. Bias, assumptions, reasons and interests in the research topic | p.8, lines 203-208  p.9, line 211 |
| **Domain 2: Study Design** |  |  |
| *Theoretical framework* |  |  |
| 9. Methodological orientation and Theory | What methodological orientation was stated to underpin the study? e.g. grounded theory, discourse analysis, ethnography, phenomenology, content analysis | p.6, lines 140-163 |
| *Participant selection* |  |  |
| 10. Sampling | How were participants selected? e.g. purposive, convenience, consecutive, snowball | p.7, line 165-166 |
| 11. Method of approach | How were participants approached? e.g. face-to-face, telephone, mail, email | p.7, lines 168-173 |
| 12. Sample size | How many participants were in the study? | p.10, line 252 |
| 13. Non-participation | How many people refused to participate or dropped out? Reasons? | p.10, lines 257-259 |
| *Setting* |  |  |
| 14. Setting of data collection | Where was the data collected? e.g. home, clinic, workplace | p.8, lines 199-201 |
| 15. Presence of non-participants | Was anyone else present besides the participants and researchers? | p.8, lines 200-201 |
| 16. Description of sample | What are the important characteristics of the sample? e.g. demographic data, date | p.10, lines 252-257 |
| *Data collection* |  |  |
| 17. Interview guide | Were questions, prompts, guides provided by the authors? Was it pilot tested? | p.8, lines 191-196 |
| 18. Repeat interviews | Were repeat interviews carried out? If yes, how many? | p.7, lines 180-198 |
| 19. Audio/visual recording | Did the research use audio or visual recording to collect the data? | p.9, lines 211-212 |
| 20. Field notes | Were ﬁeld notes made during and/or after the inter view or focus group? | p.8, lines 209-211 |
| 21. Duration | What was the duration of the interviews or focus group? | p.8, line 208 |
| 22. Data saturation | Was data saturation discussed? | p.7, line 178 |
| 23. Transcripts returned | Were transcripts returned to participants for comment and/or correction? | p.10, lines 239-241 |
| **Domain 3: Analysis and findings** |  |  |
| *Data analysis* |  |  |
| 24. Number of data coders | How many data coders coded the data? | p.9, lines 230-234 |
| 25. Description of the coding tree | Did authors provide a description of the coding tree? | -see Table 1 on p.11, line 268  -see Table 2 on p.20, line 504  -see Table 3 on p.24, line 554 |
| 26. Derivation of themes | Were themes identiﬁed in advance or derived from the data? | p.9, lines 229-230 |
| 27. Software | What software, if applicable, was used to manage the data? | p.9, line 228 |
| 28. Participant checking | Did participants provide feedback on the ﬁndings? | p.10, lines 239-241 |
| *Reporting* |  |  |
| 29. Quotations presented | Were participant quotations presented to illustrate the themes/ﬁndings? Was each quotation identiﬁed? e.g. participant number | See Results section  p.11-25 |
| 30. Data and ﬁndings consistent | Was there consistency between the data presented and the ﬁndings? | -see main headings in Results section starting on p.11, line 260.  -see main headings used in Discussion section starting on p.25, line 556 |
| 31. Clarity of major themes | Were major themes clearly presented in the ﬁndings? | -see p.11, line 261-263 |
| 32. Clarity of minor themes | Is there a description of diverse cases or discussion of minor themes? | -see p. 11, line 263-271  -see p.13, lines 272-555 |
